# Kritische Erfolgsfaktoren für die Auswahl eines IT-Serviceproviders am Beispiel der gesetzlichen Unfallversicherungen

**DOI:** 10.1365/s40702-021-00774-4

**Published:** 2021-08-18

**Authors:** Felix Hohmeister, David Rückel

**Affiliations:** 1 AIM Austrian Institute of Management GmbH, Thomas-A-Edison-Straße 2, 7000 Eisenstadt, Österreich; 2 Johannes Kepler University Linz, Altenberger Str. 69, 4040 Linz, Österreich

**Keywords:** Deutsche Gesetzliche Unfallversicherung, E‑Government, IT-Serviceprovider, Kritische Erfolgsfaktoren, Öffentlicher Sektor, Onlinezugangsgesetz, German Social Accident Insurance, E‑government, IT service providers, Critical success factors, Public sector, Online access act

## Abstract

Seit der Einführung des E‑Government Gesetzes ist das Thema in deutschen Verwaltungen omnipräsent. Die durch das Onlinezugangsgesetz (OZG) verpflichtende Bereitstellung von Online-Services erhöht den Druck auf die Verwaltungen zusätzlich. Ganzheitliche Veränderungen der Organisation sind nötig, um sich an den Bedarf der veränderten Umwelt anzupassen. Die Bürger*innen erwarten von den Verwaltungen Komfort in der Kommunikation und digitale Verarbeitung aller Anliegen wie im privaten oder beruflichen Umfeld. Eine Unterstützung bei der Umsetzung der Anforderungen des OZG bzw. des E-Governments können IT-Serviceprovider darstellen. Diese sind in der Lage, zentralisierte, skalierbare Soft- und Hardwarelösungen bereitzustellen und Defizite im vorhandenen Know-How sowie Ressourcenengpässe im öffentlichen Sektor zu kompensieren. Entscheidend dafür ist die rechtskonforme und zukunftssichere Auswahl eines IT-Serviceproviders. Dieser Forschungsbeitrag identifiziert und analysiert die für die Auswahl eines IT-Serviceproviders maßgeblichen kritischen Erfolgsfaktoren im Umfeld der gesetzlichen Unfallversicherungen Deutschlands. Aus Sicht der Wissenschaft trägt dieser Artikel somit zu einer breiteren Diskussion im Bereich Strategien zu e‑Government bzw. E‑Governance sowie Umsetzung des OZG bei. Für die Praxis wird aufgezeigt, wie ein strategischer Mehrwert aus der Neuausrichtung der gesetzlichen Unfallversicherungen hin zu einer modernen Verwaltung entstehen kann, um zukünftigen Entscheidungsträger*innen weitsichtige Entscheidungen zu ermöglichen und den Bürger*innen optimierte Services anbieten zu können.

## Abstract (en)

Since the introduction of the E‑Government Act, the topic has been omnipresent in German administrations. The availability of online services, made mandatory by the Online Access Act (OZG), adds to the pressure on administrations. Holistic changes in the organization are necessary in order to adapt to the needs of the changed environment. Citizens expect administrations to provide convenient communication and digital processing of all requests, just as they do in their private or professional lives. IT service providers can support in implementing the requirements of the OZG or e‑government. They are able to offer centralized, scalable software and hardware solutions and compensate for deficits in existing know-how and resource bottlenecks in the public sector. The decisive factor here is the legally compliant and future-proof selection of an IT service provider. This research article identifies and analyzes the critical success factors for the selection of an IT service provider in the environment of Germany’s statutory accident insurers. From an academic perspective, this article thus contributes to a broader discussion in the area of e‑government or e‑governance strategies and implementation of the Online Access Act (OZG). For practical purposes, it shows how strategic added value can arise from the reorientation of the statutory accident insurers towards a modern administration, in order to enable future decision-makers to make far-sighted decisions and to be able to offer citizens optimized services.

## Einleitung

In der Informationstechnologie wurden in den letzten Jahren erhebliche Fortschritte erzielt indem innovative Technologien entwickelt wurden. Mit dem Ziel einer digitalen Transformation wird die Digitalisierung und damit substantielle Veränderung von Prozessen, Produkten, Services und Geschäftsbeziehungen vorangetrieben (Karimi und Walter 2015, S. 69). Diese Elemente sowie die (Unternehmens-)kultur in Organisationen in Wirtschaft und Verwaltung müssen über Ländergrenzen vergleichbar und damit implizit wettbewerbsfähig sein (Hartl und Hess 2017, S. 1–3). Um diesen Strukturwandel zu beschleunigen und sicherzustellen, hat die Bundesrepublik Deutschland im Jahr 2013 das E‑Government-Gesetz als Leitfaden zur Digitalisierung von Services für die Länder und Kommunen entwickelt (EGovG 2013). Den Grundstein für die Einführung des E‑Government-Gesetzes legte davor die Europäische Union mit einem konkreten Aktionsplan. Das Onlinezugangsgesetz (OZG), als Teilbereich des E‑Governments, erhöht den Handlungsdruck auf den öffentlichen Sektor zusätzlich. Im OZG ist geregelt, dass die föderalen Strukturen ihre Onlineportale bis zum 31. Dezember 2022 zu einem Portalverbund verknüpfen und medienbruchfrei zentral bereitstellen müssen (Gesetz zur Verbesserung des Onlinezugangs zu Verwaltungsleistungen (Onlinezugangsgesetz – OZG) 2017).

Die Vorgaben des E‑Government-Gesetzes und des OZG werden seit dem Jahr 2018 auch im Spitzenverband der Gesetzlichen Unfallversicherung (DGUV) diskutiert. Die DGUV vereint insgesamt 33 Berufsgenossenschaften und Unfallversicherungsträger innerhalb Deutschlands in diesem Verband. Trotz des gemeinschaftlichen Ansatzes verfolgen die einzelnen Mitglieder des Verbandes unterschiedliche Interessen und Vorgehensweisen bei der Umsetzung des E‑Governments sowie der assoziierten Digitalisierungsbemühungen. Sie verfügen über unterschiedliche Know-how- und Ressourcenkapazitäten, um diese Themen zu behandeln und darauf zu reagieren. Es ergeben sich zahlreiche Probleme und Herausforderungen, die den Fortschritt in den Verwaltungen hemmen. Neben den externen Faktoren spielen der Mangel an Fachkräften, die historisch gewachsenen und heterogenen Hard- und Softwarestrukturen und die geringe Innovationsfreude eine entscheidende Rolle (Kühn et al. 2009, S. 100).

Dennoch erwarten Bürger*innen die zentrale Bereitstellung von Informationen sowie deren digitale Verarbeitung und Kommunikation. Unterstützung bei der Umsetzung der Anforderungen des OZG und des E‑Governments können IT-Serviceprovider leisten. Diversifizierte IT-Serviceprovider stellen zentralisierte, skalierbare Soft- und Hardwarelösungen bereit. Dies ist eine Möglichkeit, Defizite im vorhandenen Know-How sowie Ressourcenengpässe seitens der Verwaltung zu kompensieren. Entscheidend für den Erfolg der Nutzung von externen Services ist eine rechtskonforme und strategische und damit zukunftssichere Auswahl eines IT-Serviceproviders.

Die Shared-Services-Interessengemeinschaft für die gesetzliche Unfallversicherung (SIGUV) stellt einen Versuch dar, die gemeinsamen Ressourcen, Systeme und Anwendungen in einem übergreifenden IT-Serviceprovider zu bündeln. Dieser zentrale Ansatz mit 13 unterschiedlichen, gleichberechtigten Partnern hat in der Vergangenheit nicht immer funktioniert. Zu viele Partikularinteressen haben die Umsetzung von Vorhaben erschwert oder komplett verhindert. Externe etablierte Anbieter (z. B. Amazon, Google, Microsoft, SAP) mit deren Infrastruktur beziehungsweise Cloud-Technologie wurden aus politischen Entscheidungen heraus bisher nicht betrachtet.

In diesem Beitrag werden kritische Erfolgsfaktoren untersucht, die für die Auswahl eines geeigneten IT-Serviceproviders für die gesetzlichen Unfallversicherungen maßgeblich entscheidend sind. Das Ziel ist die Anforderungen der gesetzlichen Unfallversicherungen Deutschlands an IT-Serviceprovider zu identifizieren, zu analysieren und die gewonnenen Erkenntnisse in einer strategischen Handlungsempfehlung mit geeigneten Maßnahmen zu assoziieren. Die gewonnenen Erkenntnisse sollen die zukunftssichere, rechtskonforme und langfristige Auswahl eines IT-Serviceproviders für öffentliche Verwaltungen erleichtern beziehungsweise letztlich ermöglichen. Die aus dem Ziel abgeleitete Forschungsfrage für diesen Beitrag lautet entsprechend: *Welche kritischen Erfolgsfaktoren beeinflussen maßgeblich die gesetzlichen Unfallversicherungen bei der Auswahl eines IT-Serviceproviders im Kontext des E‑Governments?*

Nach einer kompakten Darstellung des Stands der Forschung und der angewendeten Forschungsmethodik wird die Ableitung der Erfolgsfaktoren beschrieben, auf welche im Anschluss detailliert eingegangen wird. Der Beitrag schließt in einer Diskussion der Ergebnisse.

## Grundlagen zur Digitalisierung im öffentlichen Sektor

Legner et al. (2017) definieren den Begriff der Digitalisierung klassisch in zwei beziehungsweise drei Auslegungen. Zum einen steht die Digitalisierung für die Überführung analoger Daten in eine digitale und elektronisch verfügbare Form. Des Weiteren werden Prozesse innerhalb der Organisationen, die vorher durch Menschen ausgeführt wurden, durch die Digitalisierung auf ein technologisch gestütztes System übertragen: dies entspricht einer IT-gestützten Automatisierung. Durch die (Teil‑)Automatisierung von Arbeitsprozessen verändern sich Arbeits- und Verhaltensweisen der Individuen und der Effizienzgewinn auf Organisationsebene rückt zunehmend in den Fokus. In der dritten Auslegung wird die Digitalisierung (ggf. fälschlich) mit dem Begriff der digitalen Transformation gleichgesetzt. Die digitale Transformation stellt jedoch einen umfassenderen Prozess dar, der signifikante Veränderungen durch den Einsatz von digitalen Technologien in Wirtschaft, Verwaltung und in der Gesellschaft hervorruft. Nach Pousttchi (2017) wird zwischen der Veränderung der Leistungserstellung, dem Leistungsangebot und der Kundeninteraktion unterschieden. Prinzipiell wird die digitale Transformation nicht nur als Prozess gesehen, sondern auch als strategische Ausrichtung eines Unternehmens, die sich grundlegend auf Produkte, Services und Geschäftsmodelle auswirkt und diese verändert. Des Weiteren führen der Einsatz von digitalen Innovationen im Rahmen der digitalen Transformation zu unternehmensweiten organisatorischen Veränderungen und zur Erschließung neuer Geschäftsmodelle (Matt et al. 2015).

Laut der Speyerer Definition von Electronic Government ist für die Durchführung von Verwaltungsaufgaben (Government) der Einsatz elektronischer Hilfsmittel und Medien (Electronic) entscheidend. Dabei gilt diese Speyerer Definition für alle Ebenen des öffentlichen Sektors, von der kommunalen bis zur Bundesebene. Generell umfasst e‑Government nicht nur die Prozesse zwischen den einzelnen Verwaltungsorganen (G2G), sondern auch zu den Bürger*innen (G2C), der Wirtschaft (G2B) und den Non-Profit-Organisationen (G2N) des dritten Sektors. Darüber hinaus steht der Technologieeinsatz im Zentrum des e‑Governments, um den Zugang zu Verwaltungsdienstleistungen den Bürger*innen beziehungsweise den Geschäftspartner*innen zur Verfügung stellen zu können (Silcock 2001). Innovative Technologien zur Bereitstellung von Verwaltungsinformationen und -dienstleistungen über das Internet spielen hierbei eine besondere Rolle (West 2004). Am 18.08.2017 trat das Onlinezugangsgesetz (OZG) in Kraft und soll den Onlinezugang zu digital angebotenen Services von Bund, Ländern und Kommunen einheitlich regeln und fördern (Gesetz zur Verbesserung des Onlinezugangs zu Verwaltungsleistungen (Onlinezugangsgesetz – OZG) 2017). Laut § 1 des OZG sollen die Verwaltungsportale der einzelnen föderalen Strukturen zu einem Portalverbund zusammengeführt werden. Die öffentlichen Träger haben bis zum 31. Dezember 2022 Zeit, ihre Portale bereitzustellen und untereinander zu verknüpfen. Damit die tatsächliche Verfügbarkeit von Services und Informationen abgebildet werden kann, hat die Europäische Union ein Reifegradmodell entwickelt. Dieses Reifegradmodell definiert sechs Stufen, um die Verfügbarkeit von Onlineverwaltungsleistungen besser messen zu können. Skaliert wird von 0 (Offline) bis 6 (Automatisierter Service), wobei 0 dafür steht, dass die Verwaltung die Services nur offline zur Verfügung stellt und 6 dafür, dass alle Services vollständig elektronisch und automatisiert ablaufen (Aguzzi et al. 2018).

Der Begriff der digitalen Innovation bezeichnet ein Produkt, einen Prozess oder ein Geschäftsmodell, das als neu wahrgenommen wird. Die von den hervorgerufenen Änderungen betroffenen Anwender*innen müssen sich daran anpassen und die IT, in der die digitale Innovation oftmals verankert ist, muss diesen Vorgang unterstützen (Fichman et al. 2014). Darüber hinaus bieten die digitalen Technologien eine Voraussetzung, um digitale Innovationen einsetzen zu können (Yoo et al. 2010). Eine der Technologien, der maßgebliches Innovationspotential im Rahmen der digitalen Transformation zugesprochen wird, ist das Cloud-Computing (Berman et al. 2012). Der Begriff Cloud-Computing subsumiert die Nutzung von digitalen Ressourcen, ohne die dafür notwendige Infrastruktur selbst zu betreiben, sondern dynamisch nach Bedarf zu mieten. Diese Technologie stellt eine Möglichkeit dar, einen schnellen Zugriff auf mit anderen Teilnehmer*innen geteilte Ressourcen (Anwendungen, Netzwerk, Computer, Speicher, Services) zu erhalten. Dabei kann zeitnah auf sich verändernde Bedingungen reagiert werden. Mit einem minimalen Verwaltungsaufwand und kaum notwendiger Interaktion des IT-Serviceproviders können Ressourcen verwaltet werden (Mell und Grance 2011). Cloudressourcen werden über verschiedene Servicemodelle zur Verfügung gestellt, üblicherweise in den Dimensionen Applikationen, Infrastruktur und/oder Plattformen. Cloud-Computing ist in der Wirtschaft weit verbreitet und wird zunehmend in der Verwaltung als Alternative zu etablierten On-Premise-Lösungen, speziell für dynamische Anwendungsfälle, im E‑Government diskutiert (Zwattendorfer et al. 2013).

Diese werden von (mitunter externen) IT-Serviceprovidern zur Verfügung gestellt. Ein IT-Serviceprovider stellt IT-Services für einen oder mehrere Kund*innen zur Verfügung. Der Begriff des IT-Serviceproviders lässt sich im Zusammenhang mit dem IT-Servicemanagement (ITSM) erfassen. ITSM vereint Methoden und Maßnahmen, um die Geschäftsprozesse bestmöglich durch die IT-Organisation zu unterstützen. Einen faktischen Standard für IT-Serviceprovider stellt hierbei das ITIL-Framework dar (Hochstein et al. 2005). Ein modernes ITSM legt den Fokus nicht mehr nur auf technikorientierte Funktionen, sondern es rücken vielmehr die Kund*innen und damit der Dienstleistungsgedanke in den Vordergrund. IT-Dienstleistungen müssen flexibel, wirtschaftlich und effizient angeboten werden (Böhmann und Krcmar 2004). Ein IT-Serviceprovider ist somit ein zentraler Bestandteil einer serviceorientierten IT-Infrastruktur. IT-Serviceprovider ermöglichen darüber hinaus, bedarfsgerecht Services zur Verfügung zu stellen und abzurechnen. Investitionen in eine unternehmenseigene, komplexe IT-Infrastruktur sind in diesem Fall nicht mehr notwendig.

## Methodisches Vorgehen

Aufgrund der geringen Dichte an wissenschaftlicher beziehungsweise praxisnaher Literatur für das Forschungsthema entschieden sich die Autor*innen für eine explorative Untersuchung, die immer dann Anwendung findet, wenn der Stand der Forschung wenig über zugrundeliegende Zusammenhänge bietet und damit erste Identifikation und Analyse von neuen Erkenntnissen im Mittelpunkt der Bemühungen stehen. Im Rahmen dieser explorativen Untersuchung wurden zwischen Mai 2020 und August 2020 insgesamt neun Expert*innen aus dem Umfeld der gesetzlichen Unfallversicherungen befragt. Die Expert*innenakquise erfolgte durch eine Schlagwortsuche in den sozialen Netzwerken LinkedIn und Xing, die Kontaktaufnahme direkt per E‑Mail oder persönlicher Empfehlungen. Im Vorfeld der explorativen Untersuchung wurden aus der Literatur die befragungsrelevanten Konstrukte abgeleitet und beschrieben. Aufgrund der fehlenden theoretischen Befundung wurden gegenstandsrelevante Phänomene beschrieben (Gläser und Laudel 2010), welche in diesen Konstrukten subsumiert wurden. Damit bildete (explorativ) das Forschungsziel und die -frage die Grundlage für die Konstrukte der definierten Problemstellung. Als konkrete Methode der durchgeführten Experteninterviews wurde das problemzentrierte Interview, als Variante des narrativen Interviews, gewählt und durch einen semistrukturierten Interviewleitfaden ergänzt. Die insgesamt 28 Fragen wurden in vier Fragenblöcken und einen generischen Einleitungs- und Abschlussteil aufgeteilt. Die Fragetypen wurden nach inhaltlichen Aspekten typisiert, sodass die Fragen den möglichst optimalen und umfassenden Output für die Beantwortung der Forschungsfrage ergeben. Der Fokus der Typisierung lag auf dem Gegenstand der Frage oder auf dem Inhalt der Frage. Die Fragen wurden vom Autor definiert und durch einen Pretest des Fragenkatalogs finalisiert. Ziel der explorativen Untersuchung war es kritische Erfolgsfaktoren zu eruieren, welche maßgeblich für den Unternehmenserfolg und die Auswahl eines IT-Serviceproviders herangezogen werden können. Der semistrukturierte Interviewleitfaden wurde danach einem Pretest mit zwei Proband*innen aus Wissenschaft und Praxis unterzogen und dadurch inhaltlich und hinsichtlich der Formulierung geschärft. Die Durchführung der Interviews erfolgte direkt (face-to-face) mittels Videokonferenz- beziehungsweise Kollaborationswerkzeugen. Die grundsätzliche Entscheidung für die fernmündliche Befragung war den zum Zeitpunkt der Befragung gültigen Covid-19-Maßnahmen geschuldet, die Wahl des konkreten Werkzeugs (z. B. Zoom, MS Teams, Skype) den jeweiligen Vorlieben oder Vorgaben der Expert*innen. Die erhobenen qualitativen Daten wurden nach einer vollständigen Transkription der Interviews durch eine qualitative Inhaltsanalyse nach Mayring (2010) ausgewertet. Die inhaltstragenden Textstellen in den Transkripten wurden zunächst kodiert, zusammengefasst und auf ein einheitliches sprachliches Niveau gehoben. Anschließend führte der durchführende Interviewer die Paraphrasierung und Reduktion des Textes durch. Eine abschließende zweite Reduktion verkürzte die Auswertung um inhaltsgleiche Stellen. Die so gewonnenen finalen Paraphrasen bildeten die Grundlage für die weitere Analyse.

## Ableitung der kritischen Erfolgsfaktoren

Kritische Erfolgsfaktoren werden bestimmten unternehmensspezifischen Bereichen, die maßgeblich für den Unternehmenserfolg verantwortlich sind, zugeordnet und mit entsprechend definierten Ergebnissen verknüpft (Boynton und Zmud 1984). Die Definition und Einhaltung kritischer Erfolgsfaktoren bilden die Grundlage für den fortwährenden Erfolg eines Unternehmens. Hierdurch kann die Performance dieser Bereiche durch die strategischen Verantwortlichen des Unternehmens besser überwacht werden und die Unternehmensstrategie kann an die technologischen Bedürfnisse des Betriebs adaptiert werden. Werden die Ergebnisse in den definierten Bereichen erfüllt, ergibt sich für das Unternehmen ein bedeutender technologischer Wettbewerbsvorteil (Forster und Rockart 1989). Die Definition der kritischen Erfolgsfaktoren hat für die öffentlichen Verwaltungen die gleiche Semantik wie für die Privatwirtschaft.

Konkrete Definitionen kritischer Erfolgsfaktoren für die Auswahl eines IT-Serviceproviders stellen im Umfeld der gesetzlichen Unfallversicherungen eine komplexe und politische Angelegenheit dar. Dies liegt an der Vielfalt verschiedener Interessensgemeinschaften aus historisch gewachsenen organisatorischen und technischen Strukturen sowie aus Einflüssen durch die Gesetzgebung, durch Softwarehersteller und durch das übergreifende E‑Government.

Das Ergebnis der explorativen Untersuchung zeigt, dass jeder Unfallversicherungs-Träger (UV-Träger) eindeutige individuelle Anforderungen daran festmacht, welche Bedingungen er an einen IT-Serviceprovider stellt und welche kritischen Erfolgsfaktoren erfüllt sein müssen, um den Produktivbetrieb zu jedem Zeitpunkt sicher, datenschutzkonform und performant zu garantieren. Interne kritische Erfolgsfaktoren stellen Maßnahmen und Handlungsmöglichkeiten der gesetzlichen Unfallversicherungen dar, die den sicheren Betrieb und die Auswahl eines IT-Serviceproviders maßgeblich beeinflussen. Durch sie können die vorhandenen Herausforderungen mittels der Erfüllung der Faktoren bewältigt werden. Externe kritische Erfolgsfaktoren hingegen müssen erfüllt sein, damit ein IT-Serviceprovider für gesetzliche Unfallversicherungen gewählt werden kann. Alle hier erfassten kritischen Erfolgsfaktoren sind für die Sicherstellung des Produktivbetriebes und für die Auswahl eines IT-Serviceproviders entscheidend. Die Ergebnisse der gegenständlichen explorativ-empirischen Forschung zeigen, dass sich bestimmte kritische Erfolgsfaktoren in den Aussagen der Expert*innen häufen und dass eine besondere Priorität auf diesen liegt. Alle erhobenen kritischen Erfolgsfaktoren wurden anhand einer Frequenzanalyse (Häufigkeitsanalyse) weiter verdichtet und strukturiert. Die neun, auf Basis der Frequenzanalyse identifizierten und priorisierten kritischen Erfolgsfaktoren, können somit als maßgeblich bezeichnet werden.

## Kritische Erfolgsfaktoren für die Auswahl von IT-Serviceprovider

**Abb. 1:**
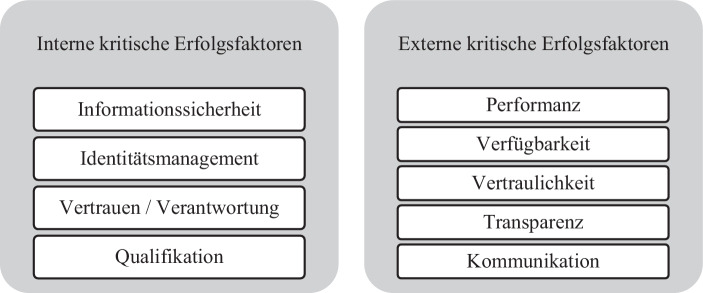
Interne und externe kritische Erfolgsfaktoren. (Eigene Darstellung)

In diesem Kapitel werden die durch die Interviews erfassten maßgeblichen kritischen Erfolgsfaktoren näher beschrieben und mit passenden Maßnahmen zur Erfüllung dieser ergänzt. Die neun aus insgesamt 25, durch die Frequenzanalyse identifizierten, maßgeblichen internen und externen kritischen Erfolgsfaktoren sind in Abb. 1 dargestellt.

### Interne Erfolgsfaktoren

#### Informationssicherheit

Der Umgang mit Sozialdaten hat zur Folge, dass gesetzliche Unfallversicherungen besonders strenge gesetzliche Vorgaben des Sozialgesetzbuchs und geltende Datenschutzbestimmungen einhalten müssen. Dementsprechend hat die Erfüllung dieser Vorgaben eine hohe Priorität. Eine Verletzung des Datenschutzes kann sowohl für den Betroffenen als auch für die gesetzlichen Unfallversicherungen erhebliche Folgen haben. Die Wahrung des Datenschutzes und die sichere Konfiguration der Systeme durch technische Verfahren muss für interne Prozesse kongruent zu den erbrachten Dienstleistungen eines IT-Serviceproviders gelten. Der empirisch erfasste Stellenwert der Informationssicherheit deckt sich mit den Angaben aus der Literatur (Büllingen et al. 2009).

Um den Erfolgsfaktor Informationssicherheit zu realisieren, ist ein proaktiver Einsatz von organisatorischen und technischen Verfahren des IT-Serviceproviders (z. B. Patchmanagement) sowie die Überprüfung der eingesetzten internen und externen Produkte und Prozesse auf ihre Datenschutzkonformität zwingend erforderlich. Außerdem müssen technischen Verfahren zum Schutz der Daten (z. B. Verschlüsselung bereits auf Datenbankebene) angewendet und die Auswahl eines europäischen IT-Serviceproviders an die Datenschutzgrundverordnung gebunden sein.

#### Identitätsmanagement

Die Vorgaben des E‑Governments und des OZG erfordern die zentrale Bereitstellung von digitalen Dienstleistungen sowie die Vernetzung der Verwaltungen auf Bundes‑, Landes- und Kommunalebene. Für die Bereitstellung sind sichere Authentifizierungsverfahren und Berechtigungskonzepte notwendig. Ein/e Interviewpartner*in verwies auf die nicht vorhandene Authentifizierung innerhalb des OZG, weshalb aus Sicht des/der Interviewpartner*in das OZG „scheitern werde“. Eine zukünftige Auswahl eines IT-Serviceproviders ist demnach an die Erfüllung dieses kritischen Erfolgsfaktors gebunden. Die Erkenntnisse und kritischen Erfolgsfaktoren decken sich mit den Aussagen in der Literatur (Hommel 2007).

Die Verwirklichung des Erfolgsfaktors Identitätsmanagement ist nur durch digitale Innovationen wie beispielsweise die Einführung von Technologien analog zu jenen der digitalen Wallets möglich und die dadurch durchgängige Authentifizierung von Benutzern. Übergreifende Konzepte zur zentralen Vernetzung der Verwaltungen, Systeme und digitalen Services müssen initiiert werden.

#### Vertrauen und Verantwortung

Faktoren wie Vertrauen und Verantwortung bestimmen über den Erfolg oder den Misserfolg einer Organisation oder eines Vorhabens. Mitarbeiter*innen und Führungskräfte müssen Verantwortung für ihre Handlungen übernehmen, sich intrinsisch motivieren und Ziele der Organisation verfolgen. Dies geschieht bisher in geringem Maß und einige wenige Leistungsträger bringen die Organisation langsam voran. Ein Grundvertrauen muss durch eine Transparenz der Prozesse, Workflows und Entscheidungen gewährleistet werden und zwischen den Mitarbeiter*innen, aber auch zwischen dem Kunden*innen und Dienstleistern bestehen. Diese zwei Grundsätze gelten in einem gleichen Maß für die Auswahl eines IT-Serviceproviders. In der Literatur werden der vertrauensvolle Umgang mit den Mitarbeiter*innen und die Bereitschaft zur Übernahme von Verantwortung ebenfalls als kritische Erfolgsfaktoren gesehen (Gazdar 2006).

Der Erfolgsfaktor Vertrauen lässt sich nur durch eine offene und ehrliche Kommunikation aller Parteien und transparenter interner und externer Prozesse und Workflows einhalten. Die Übernahme der Verantwortung der Mitarbeiter*innen kann nur durch eindeutige Anforderungen in allen organisatorischen und technischen Bereichen erfüllt werden.

#### Qualifikation

Die Qualifikation der Mitarbeiter*innen und das vorhandene Know-how über die bereitgestellten Services sind essenziell, um den produktiven langfristigen Betrieb und den Erfolg sicherzustellen. Laut einer/m Interviewpartner*in ist es dabei irrelevant, ob ein System inhouse oder durch einen IT-Serviceprovider betrieben wird. Erschwerend kommt bei der Erfüllung dieses Faktors hinzu, dass der öffentliche Dienst für Mitarbeiter*innen unattraktiv ist und dass Know-how durch den demografischen Wandel und durch die Verlagerung von Systemen erneut verloren geht. Es ist schwer neue qualifizierte Fachkräfte für den öffentlichen Sektor begeistern und vorhandene gut ausgebildete Leistungsträger halten zu können. In der Literatur werden die Erfolgsfaktoren Qualifikation und Kompetenzentwicklung als besondere strategische Faktoren herausgestellt (Boes et al. 2012).

Der Erfolgsfaktor Qualifikation kann nur durch die Nutzung von Synergieeffekten und Know-how anderer UV-Träger und Mitgliedsunternehmen erreicht werden. Bei der Verlagerung von Infrastruktur und Systemen ist ein interner Experte zu erhalten, der sich mit den verlagerten Services auskennt. Initiierung von attraktiven Schulungs- und Förderungskonzepten für interne Mitarbeiter*innen und eine umfassende Dokumentation und Erfahrungssicherung stellen eine Voraussetzung dar für die Erfüllung dieser Erfolgsfaktoren.

### Externe Erfolgsfaktoren

#### Performanz/Verfügbarkeit

Hohe Anforderungen der versicherten Betriebe, der internen und externen Kund*innen, der Mitarbeiter*innen sowie der Gesetzgebung an zentral bereitgestellte Services müssen erfüllt werden. Schwerpunktthemen sind hierbei neben dem Datenschutz und der Informationssicherheit die performante Anbindung und Hochverfügbarkeit der Services. Ein/e Interviewpartner*in verwies darauf, dass heute die gleichen hohen Anforderungen an die Performanz und Verfügbarkeit der Services gestellt werden, wie sie aus dem digitalen privaten Umfeld bekannt sind. Das Nutzererlebnis und die Anwenderzufriedenheit mit dem angebotenen Service gewinnen zunehmend an Priorität. In der Literatur gelten die beiden kritischen Erfolgsfaktoren, gerade bei der Bereitstellung von Services, als sehr wichtig (BITKOM 2010).

Eine Maßnahme zur Realisierung der kritischen Erfolgsfaktoren Performanz und Verfügbarkeit stellt die Definition von Service Level Agreements (SLAs) mit dem IT-Serviceprovider und die Schaffung von performanten Verbindungen zwischen den Rechenzentren und den Standorten der gesetzlichen Unfallversicherungen sowie dem IT-Serviceprovider beziehungsweise Plattformanbieter dar. Außerdem sollten etablierte, marktfähige und wirtschaftlich stabile IT-Serviceprovider ausgewählt werden.

#### Transparenz/Vertraulichkeit

Große IT-Serviceprovider legen ihre internen Prozesse oft nicht offen. Die Folge davon können Datenabflüsse in Drittländer und unklare Vorgehensweisen bei der Speicherung und Verarbeitung von sensiblen Sozialdaten sein. Es ist daher essenziell, dass die Transparenz der in Anspruch genommenen Services und Technologien eines IT-Serviceproviders erfüllt sind. Die Literatur unterstützt die gewonnenen Erkenntnisse (Picot et al. 2011).

Die Implementierung von ISO- oder BSI-Zertifikaten des IT-Serviceproviders gilt als Gütesiegel, die die Transparenz und Vertraulichkeit der internen Prozesse gewährleistet. Es gilt darüber hinaus kleinere IT-Serviceprovider mit eindeutigen offenen Strukturen oder alternativ einen internen IT-Serviceprovider einzusetzen. Der Kauf beziehungsweise die Übernahme des einzusetzenden Dienstleisters stellt darüber hinaus eine Option dar, jedoch müssen stets die Kosten dem Nutzen gegenübergestellt werden.

#### Kommunikation

Ein IT-Serviceprovider muss mit allen nötigen Informationen versorgt werden, um das Business der gesetzlichen Unfallversicherungen und der einzelnen UV-Träger bestmöglich betreuen zu können. Umgekehrt muss ein IT-Serviceprovider ebenfalls vollumfänglich kommunizieren und Entscheidungen zielgerichtet treffen. Es müssen zentrale Ansprechpartner*innen bereitgestellt werden und eindeutige Zuständigkeiten definiert sein. Sind die Kommunikationswege blockiert oder Anforderungen unklar, so gilt dieser kritische Erfolgsfaktor als nicht erfüllt. Die Kommunikation stellt für den Unternehmenserfolg somit einen zentralen Bestandteil dar (Rademacher 2007).

Die Initiierung zentraler Ansprechpartner*innen mit einem entsprechenden Know-how und die Einführung eines zentralen und einflussreichen Chief Information Officer (CIO) mit umfassenden Entscheidungsbefugnissen stellen Maßnahmen zur Erfüllung des kritischen Erfolgsfaktors Kommunikation dar. Die Einhaltung definierter Maßnahmen zur Erfüllung der kritischen Erfolgsfaktoren und die Erkenntnisse aus der strategischen Handlungsempfehlung können eine erfolgreiche, rechtskonforme und zukunftssichere Auswahl eines IT-Serviceproviders ermöglichen.

## Interpretation der Ergebnisse

Im öffentlichen Sektor und insbesondere innerhalb der gesetzlichen Unfallversicherungen wurden aktuelle Themen wie die Digitalisierung, der Einsatz von IT-Serviceprovidern und neue Vorgaben durch das e‑Government bisher nicht intensiv behandelt. Innerhalb der gesetzlichen Unfallversicherungen hat sich über viele Jahre ein inhomogenes Infrastruktur- und Anwendungsgeflecht ergeben, das nun Probleme bei der Überführung in neue digitale Strukturen erzeugt. Ebenso inhomogen sind der der Digitalisierungsfortschritt und die Haltung zum Einsatz von IT-Serviceprovidern zwischen den einzelnen UV-Trägern. Zusätzlich zu den ohnehin strukturellen Problemen des öffentlichen Sektors sind die gesetzlichen Unfallversicherungen durch große politische Spannungen und einen ständigen Wettstreit um die Durchsetzung spezifischer Partikularinteressen gegenüber dem Gemeinwohl geprägt. Es fehlt der Wille, gemeinsame Vorhaben voranzutreiben, Innovationen zu fördern und Synergieeffekte zu erzeugen. Ein fehlendes Know-how, unzureichende Ressourcen und langsame Prozesse erschweren den Fortschritt zusätzlich. Zukünftig können sich die verschiedenen Ökosysteme der gesetzlichen Unfallversicherungen dezentral organisieren, aber sich dabei zentral am gleichen Kundenversprechen orientieren. Innovative Technologien und Anforderungen werden nur noch durch interne und externe Kooperationen wirtschaftlich umsetzbar sein. Unumgänglich ist somit die Bildung einer zentralen IT und die zukünftige Rationalisierung von standardisierten IT-Tätigkeiten. Interne IT-Serviceprovider werden zwar teilweise genutzt, jedoch sind zu viele Parteien bei der oft verzögerten Entscheidungsfindung involviert und Anforderungen bleiben unklar oder werden nicht kommuniziert. Vorhandene gemeinschaftliche IT-Leistungen und Shared-Services-Anbieter werden als überladen, langsam und unflexibel empfunden. Ein hoher zeitlicher Druck, der durch das OZG entsteht, stellt viele UV-Träger vor eine überfordernde technologische und organisatorische Aufgabe bei der Umsetzung der gesetzlichen Vorgaben. Externe IT-Serviceprovider können durch Gesetzesänderungen inzwischen auch für die gesetzlichen Unfallversicherungen genutzt werden. Es gilt, gemeinsam zu klären, in welchem Umfang diese Anbieter zum Einsatz kommen. Externe IT-Serviceprovider werden oft nicht als mögliches Potenzial, sondern vielmehr als Bedrohung wahrgenommen. Interne individuelle Lösungen und Servicekonzepte werden häufig etablierten externen Produkten vorgezogen.

Dieses Forschungsprojekt umfasste nicht die Definition von messbaren Key Performance Indicators (KPIs). Es ergibt sich somit ein zusätzliches Forschungsfeld, die definierten maßgeblichen kritischen Erfolgsfaktoren in einer folgenden Forschungsarbeit durch die Bestimmung konkreter KPIs und durch die Realisierung eines Konzeptes zur Überwachung dieser weiter zu erforschen. Es bedarf außerdem weiterer Untersuchungen darüber, inwiefern die strategische Handlungsempfehlung umgesetzt wird und welche Auswirkungen diese auf die Gesamtunternehmung der gesetzlichen Unfallversicherungen hat. Die erarbeiteten kritischen Erfolgsfaktoren sollten in einem nächsten Schritt von weiteren Stakeholdern evaluiert und verfeinert werden. Eine Studie, die den zukünftigen Umgang mit IT-Serviceprovidern, innovativen Technologien, kritischen Erfolgsfaktoren und technologischen sowie organisatorischen Veränderungen innerhalb der gesetzlichen Unfallversicherungen erforscht, würde diese Forschungsarbeit gut ergänzen.
